# Editorial: Rising stars in infectious agents and disease: 2021

**DOI:** 10.3389/fmicb.2022.1101964

**Published:** 2022-12-20

**Authors:** François Meurens, Axel Cloeckaert

**Affiliations:** ^1^INRAE, Oniris, BIOEPAR, Nantes, France; ^2^Department of Veterinary Microbiology and Immunology, Western College of Veterinary Medicine, University of Saskatchewan, Saskatoon, SK, Canada; ^3^INRAE, Université de Tours, UMR, ISP, Tours, France

**Keywords:** viruses, vaccine, ASFV, PRRSV, VAED, TFP

Among the different pathogenic microorganisms described in animals and humans, viruses show huge diversity. Their complex interactions with their host are often challenging to study, thus limiting the development of prophylactic and therapeutic treatments.

The emergence of African swine fever (ASF) in China in 2018 has placed an increasing economic burden on many countries. The virus (ASFV) responsible for this disease was identified in Kenya in 1921 (Eustace Montgomery, 1921) but so far no vaccines have been released to the market to combat it. Many strains of ASFV have been described and this large DNA virus has developed complex interactions with innate and adaptive immune systems. Another virus responsible for major economic losses in pig production is the porcine reproductive and respiratory syndrome virus (PRRSV), which also represents a challenge for vaccine development (Lunney et al., 2016). Indeed, there are a plethora of constantly evolving PRRSV strains that vaccines would need to protect against.

Once safe and effective vaccines have been developed, the story is not over, as surveillance is needed to identify and monitor possible adverse reactions in vaccinated subjects. In a few situations, vaccine-mediated immune response may lead to exacerbated pathology upon subsequent infection caused by the targeted pathogen. This phenomenon, known as vaccine-associated enhanced disease (VAED) (Huisman et al., 2009; Munoz et al., 2021), has been reported for vaccine candidates against several viruses, such as respiratory syncytial virus (RSV), measles virus (MV), dengue virus (DENV), human immunodeficiency virus (HIV), feline immunodeficiency virus (FIV), severe acute respiratory syndrome coronavirus 1 (SARS-CoV-1), and Middle East respiratory syndrome coronavirus (MERS-CoV). In some other situations, vaccines are not available, and for some viral diseases antiviral medications are needed. Currently, few approved antiviral drugs are available (Adamson et al., 2021; Kausar et al., 2021) and research aimed at proposing and validating new ones is urgently needed.

The articles featured in this Research Topic dedicated to “*Rising stars in infectious agents and disease: 2021*” all involved one or two rising stars. Recognizing future leaders in the field of infectious agents and disease is crucial for safeguarding tomorrow's driving force in innovation. Ayanwale et al. (rising star Dr. Ferdinand Roesch) present the latest knowledge about the complex interplay between ASFV and innate immunity and its impact on viral pathogenesis. Ultimately, research studies deciphering the complex mechanisms of AFSV pathogenesis may facilitate the development of safe and effective live-attenuated vaccines against this terrible disease. This topic is currently hot and reviews are needed to keep everyone on track. Then, Proctor et al. (rising star Dr. Tobias Käser) investigated the vaccine efficacy and immunogenicity of a modified live virus (Prevacent^®^ PRRS MLV vaccine) against four heterologous type 2 PRRSV (PRRSV-2) strains in 60 pigs, and showed that Prevacent elicits various degrees of efficacy and immunogenicity against these four phylogenetically distant strains.

After these two articles dealing with pig viruses, we move to other animal models and humans and the complex question of VAED. In their review, Bigay et al. (rising star Dr. Pauline Maisonnasse) present the mechanisms that may be associated with VAED risk and that have to be taken into consideration when assessing vaccine safety and searching for ways to define models and immunization strategies for alleviating such concerns. Predicting VAED is clearly challenging, and the authors highlight various research areas that can be improved to better deal with this issue. Then, with regard to antiviral drugs, Mao et al. (rising stars Dr. Chen and Dr. Sheng) suggest the potential use of the antipsychotic drug trifluoperazine (TFP) against viruses such as vesicular stomatitis virus (VSV) and herpes simplex virus type 1 (HSV-1). They identified that TFP can inhibit virus replication through a protein kinase R (PKR)-like endoplasmic reticulum kinase (PERK)-eukaryotic initiation factor 2α (eIF2α) axis.

Together these original review articles represent significant contributions to the infectious agents and disease field, demonstrating that the new generation of scientists is ready to shine in our fascinating field of research.

## Author contributions

Both authors listed have made a substantial, direct, and intellectual contribution to the work and approved it for publication.
